# Molecular architecture of the ErbB2 extracellular domain homodimer

**DOI:** 10.18632/oncotarget.2713

**Published:** 2015-01-30

**Authors:** Shi Hu, Yuna Sun, Yanchun Meng, Xiaoze Wang, Weili Yang, Wenyan Fu, Huaizu Guo, Weizhu Qian, Sheng Hou, Bohua Li, Zihe Rao, Zhiyong Lou, Yajun Guo

**Affiliations:** ^1^ International Joint Cancer Institute & Translational Medicine Research Institute, the Second Military Medical University, Shanghai, 200433, P.R. China; ^2^ National Laboratory of Macromolecules, Institute of Biophysics, Chinese Academy of Science, Beijing, 100101, P.R. China; ^3^ Laboratory of Structural Biology and MOE Laboratory of Protein Science, School of Medicine, Tsinghua University, Beijing, 100084, P.R. China; ^4^ Cancer Center, Chinese PLA General Hospital & Chinese PLA Medical School, Beijing, 100853, P.R. China; ^5^ School of Medicine, Nankai University, Tianjin, 300071, P.R. China; ^6^ National Engineering Research Center for Antibody Medicine and Shanghai Key Laboratory of Cell Engineering & Antibody, Shanghai, 201203, P.R. China; ^7^ School of Pharmacy, Liaocheng University, Liaocheng, Shandong, 252000, P.R. China

**Keywords:** ErbB2, dimerization, signal transduction, crystal structure, Oncogene

## Abstract

Human epidermal growth factor receptors (HERs or ErbBs) play crucial roles in numerous cellular processes. ErbB2 is a key member of ErbB family, and its overexpression is recognized as a frequent molecular abnormality. In cancer, this overexpression correlates with aggressive disease and poor patient outcomes. Dimer-dependent phosphorylation is a key event for the signal transduction of ErbBs. However, the molecular mechanism of the dimerization of ErbB2 remains elusive. In the present work, we report the homodimer architecture of the ErbB2 extracellular domain (ECD) which is unique compared with other dimer-models of ErbBs. The structure of the ErbB2 ECD homodimer represents a “back to head” interaction, in which a protruding β-hairpin arm in domain II of one ErbB2 protomer is inserted into a C-shaped pocket created by domains I–III of the adjacent ErbB2 protomer. This dimerized architecture and its impact on the phosphorylation of ErbB2 intracellular domain were further verified by a mutagenesis study. We also elucidated the different impacts of two clinically administered therapeutic antibodies, trastuzumab and pertuzumab, on ErbB2 dimerization. This information not only provides an understanding of the molecular mechanism of ErbBs dimerization but also elucidates ErbB2-targeted therapy at the molecular level.

## INTRODUCTION

The epidermal growth factor receptor (EGFR) family includes four members: the human epidermal growth factor receptor 1-4(HER1-4, ErbB1-4). The overexpression and dysfunction of ErbBs result in cancer, diabetes, immune deficiencies, cardiovascular diseases, and other human diseases [1]. Dimierziation of the extracellular domains (ECDs) activates the phosphorylation of the intracellular domains (ICD)s are the key events responsible for the signal transduction of ErbBs. To date, there are 11 ligands were identified to bind to ErbBs [2]. The current model of ligand-induced ErbB dimerization is proposed to be facilitated by the shift between a ‘tethered’ intramolecular conformation and a dimerization-competent conformation. In the apo forms of ErbB1, ErbB3, and ErbB4, a protruding of domain II (designated as the ‘dimerization arm’) is buried within domain IV of the same molecule. In the dimerization-competent conformations, the dimerization arm and an adjacent loop are exposed to allow contact between the ECDs of two monomers [3, 4]. However, ErbB2 is not likely to have a ligand, and the ‘tethered’ intramolecular conformation is absent [5, 6]. Previous results have revealed that ErbB2-ErbB1/3/4 heterodimers are the most preferred [7–9], suggesting that ErbB2 is likely to be in a constitutively activated configuration that can form signaling-active heterodimers (or homodimers) without a ligand. However, it is not constitutively active when expressed at physiologically relevant levels in insect cells [10]. Moreover, a mutagenesis study [11] has shown that some mutations in domain II of the ErbB2 ECD, analogous to those ErbB1 mutants [12] that completely eliminated ErbB1 homodimerization, did not affect ErbB2/ErbB3 heterodimerization. Taken together, these results suggest that ErbB2 dimerization is different than the dimerization model for ErbB1 ECD.

## RESULTS

### Overall structure

Our interest in characterizing the architecture of the ErbB2 dimer prompted us to further study the structure and mechanism of the ErbB2 ECD homodimer. We crystallized ErbB2 ECD in complex with the Fab fragment of an anti-domain I antibody (Supplementary Text), which helps to stabilize ErbB2 ECD without affecting the dimerization arm in domain II, and solved its structure at 3.1 Å resolution in the space group *P1* (Table 1).

**Table 1:** Data collection and refinement statistics

Parameters	ErbB2 homodimer
**Data collection statistics**	
Cell parameters	*a* = 84.7 Å, *b* = 104.2 Å, *c* = 116.7 Å *α* = 107°, *β* = 99°, *γ* = 111°
Space group	*P1*
Wavelength used (Å)	1.0000
Resolution (Å)	50.0 (3.15)^c^ – 3.1
No. of all reflections	217,875
No. of unique reflections	60,521
Completeness (%)	97.7 (98.2)
Average I/σ(I)	23.1(3.8)
R_merge_^a^ (%)	10.4 (56.4)
**Refinement statistics**	
No. of reflections used (σ(F) > 0)	60,460
R_work_^b^ (%)	23.2
R_free_^b^ (%)	26.9
r.m.s.d. bond distance (Å)	0.011
r.m.s.d. bond angle (º)	1.719
Average overall B-value (Å^2^)	78.7
Ramachandran plot	
Res. in most favored regions	1679 (85.6%)
Res. in additionally allowed regions	266 (13.6%)
Res. in outlier region	17 (0.9%)

a *R_merge_* = Σ_h_Σ_l_ | I_ih_–< I_h_ > |/Σ_h_Σ_I_ < I_h_ >, where < I_h_ > is the mean of multiple observations I_ih_ of a given reflection h.

b *R*_work_ = Σ||F_p_(obs)|−|Fp(calc)||/Σ|F_p_(obs)|; *R_free_* is an R-factor for a selected subset (5%) of reflections that was not included in prior refinement calculations.

c Numbers in parentheses are corresponding values for the highest resolution shell (2.5–2.4 Å).

There are two ErbB2 ECD-Fab complex molecules in one asymmetric unit. The overall architecture of ErbB2 ECD in this complex is very similar to other reported structures of ErbB2 ECD with a root-mean-square deviation (r.m.s.d) of 1.4 Å for the Cα atoms of all residues in the ErbB2 ECD. However, an obvious shift can be observed in the dimerization arms (residues 245 to 266) in the two ErbB2 molecules.

**Figure 1:**
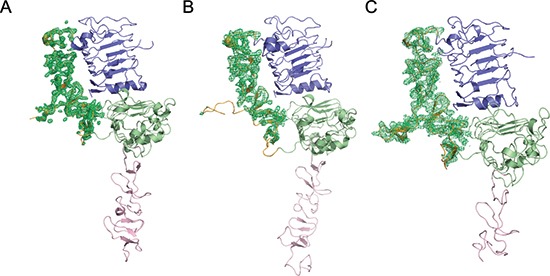
Electron density variations of the ErbB2 domain II in different forms Poor electron density of domain II can be observed in ErbB2 monomer from PDB ID code 1N8Y **(A)** and crystallographic trimer from PDB ID code 3N85 **(B)** but excellently clear density can be observed in ErbB2 dimer structure in our report **(C)**. Domains I, II, III, and IV in ErbB2 are colored slate, orange, green, and pink, respectively.

**Figure 2:**
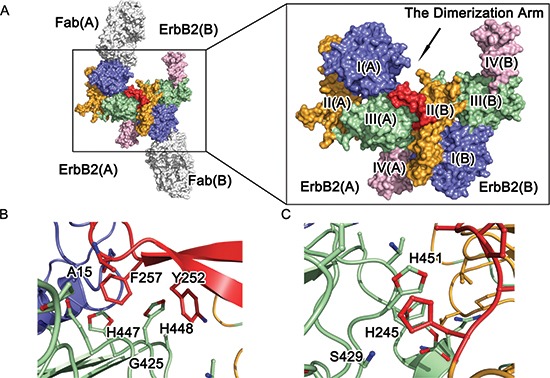
Molecular architecture of ErbB2 ECD homodimer **(A)** Crystal structure of dimeric ErbB2-Fab. Domains I, II, III, and IV of ErbB2 ECD protomer are colored blue, orange, green, and pink, respectively. The antibody is obscured, and the dimerization arm is colored red for emphasis. **(B)** and **(C)** A detailed view of the dimeric interface of the ErbB2 ECD homodimer. The side chains of the interacting residues are shown as colored sticks.

Interestingly, the dimerization arm of the ErbB2 protomer B [ErbB2(B)] is well stabilized (Fig. 1) and accommodated by a C-shaped pocket formed by domains I(A), II(A), and III(A) of the ErbB2 protomer A [ErbB2(A)] (Fig. 2A), suggesting a novel “back to head”dimeric interaction in the ErbB2 homodimer.

### ErbB2-ErbB2 interactions

ErbB2 dimerization was facilitated mostly by interactions between domain II(B) of ErbB2(B) and domains I(A) and III(A) of ErbB2(A). The dimerization arm formed a β-hairpin in the middle, which protrudes from the domain II(B) globule. Six residues (Y252 to F257) at the tip of the dimerization arm served as a “hand” to interact with the groove made by domain I(A) and domain III(A) (Fig. 2B, 2C, and S1A). An extensive network of intermolecular interactions at the receptor-receptor interfaces were composed of a number of discontinuous segments of the two neighboring ErbB2 molecules, including the domain I(B) and II(B) residues N154, Q156, H235-F236, L244-F257, V286-G287, and P295-L295 and the domain I(A) and III(A) residues L13-P17, P356, S391-P398, Q424-W430, H447-H451, and A475-Q491 (Fig. S1B, C and D, Table 2). The majority of these interactions result from the interaction of the dimerization “hand” of domain II(B) interacting with its receiving pocket composed of domain I(A) and domain III(A) (Fig. 2B and S1B), the region of domain II(B) containing the projection arm, and the shoulder of domain II(B) lying on the bench made by domain III(A) (Fig. 2C, S1C and S1D).

**Table 2:** Complete list of interactions of ErbB2 homodimer interface (≤ 4Å)

ErbB2(A)		ErbB2(B)			Distance (Å)
Residue	Atom	Residue	Atom	Domain	
	C^β^	Phe257	C^ε1^		3.94
Ala15	O	Phe257	C^δ1^	II	3.95
	O	Phe257	C^β^		3.96
Pro356	C^γ^	Thr254	C^γ2^	II	3.97
Pro398	C^β^	Leu244	C^δ2^	II	3.69
	N^ε2^		C^ε1^		3.28
	N^ε2^		C^ζ^		3.39
Gln424	C^δ^	Phe257	C^ε1^	II	3.69
	C^δ^		C^ζ^		3.44
	C^β^		C^ζ^		3.94
Gly425	N	Phe257	C^ε2^	II	3.86
	O^γ^	Leu244	C^δ2^		3.65
Ser429	O^γ^	His245	N^ε2^	II	3.88
	O^γ^	His245	C^δ2^		3.44
Trp430	O	Leu244	C^ζ3^	II	3.89
	C^ε1^		C^δ2^		3.81
	C^ε1^		C^ε2^		3.55
His447	C^ε1^	Phe257	C^ζ^	II	3.59
	C^ε1^		C^ε1^		3.91
	N^ε2^	Phe257	O		3.69
	N^ε2^	Tyr252	C^δ1^		3.90
	N^ε2^	Tyr252	C^ε1^		3.68
	C^δ2^	Phe257	C^δ2^		3.79
	C^δ2^	Tyr252	C^γ^		3.53
	C^δ2^	Tyr252	C^δ2^		3.59
His448	C^δ2^	Tyr252	C^ε2^	II	3.53
	C^δ2^	Tyr252	C^ζ^		3.39
	C^δ2^	Tyr252	C^ε1^		3.31
	C^δ2^	Tyr252	C^δ1^		3.38
	C^γ^	Phe257	C^δ2^		3.51
	C^β^	Phe257	C^ε2^		3.55
	C^β^	Phe257	C^δ2^		3.58
Thr450	O^γ1^	Ala248	C^β^	II	3.86
	C^ε1^	Ala248	C^β^		3.94
	C^ε1^	His245	C^ε1^		3.42
	C^ε1^	His245	N^δ1^		3.51
H451	N^ε2^	Ala248	C^β^	II	3.75
	N^ε2^	Ala248	N		3.97
	N^ε2^	His245	N^δ1^		3.36
	N^ε2^	Cys246	O		3.43
	C^δ2^	His245	N^δ1^		3.60
	C^δ2^	His245	C^γ^		3.84
	N^δ1^	His245	N^ε2^		3.93
	N^δ1^	His245	C^ε1^		3.52
	N^δ1^	His245	N^δ1^		3.80
	C^γ^	His245	C^ε1^		3.93
	C^γ^	His245	N^δ1^		3.87
	O^δ2^	Leu295	C^δ2^		3.29
Asp480	O^δ2^	Leu295	C^γ^	II	3.91
	O^ε1^	Ser288	O^γ^		2.90
	O^ε1^	Ser288	C^β^		3.22
	O^ε1^	Val286	C^γ1^		3.36
Glu481	O^ε1^	Val286	C^γ2^	II	3.88
	C^δ^	Val286	C^γ1^		3.60
	C^δ^	Val286	C^γ2^		3.94
	O^ε2^	Val286	C^γ1^		3.88
	O^ε2^	Val286	C^γ2^		3.46
	C^δ2^	Gln156	N^ε2^		3.58
	C^δ2^	Gln156	C^δ^		3.79
	C^δ2^	Gln156	O^ε1^		3.40
His490	N^ε2^	Gln156	N^ε2^	II	3.69
	N^ε2^	Gln156	C^δ^		3.55
	N^ε2^	Gln156	O^ε1^		2.82
	C^ε1^	Gln156	C^δ^		3.95
	C^ε1^	Gln156	O^ε1^		3.40

A set of “face to face” parallel hydrophobic π interactions can be observed: the first is between F257 in domain II(B) and H447 in domain III(A), and the second involves the phenol ring of Y252 in domain II(B) and the imidazole ring of H448 in domain III(A) (Fig. S2A, and B). Additionally, Q424 of domain III(A) participates in an intramolecular hydrogen bond with H447 of domain III(A), helping to stabilize the direction and position of the imidazole ring of H447. Additionally, nitrogen ε2 in the imidazole ring of H448 in domain III(A) forms a side-chain to main-chain hydrogen bond to the oxygen atom of F257 in domain II(B).

Another set of “face to face” parallel hydrophobic π bonds can be observed in the interactions between H245 in domain II(B) and H451 in domain III(A) and T450 in domain III(A) with A248 in domain II(B). These interactions properly fixed the position of the imidazole ring of H451 in domain III(A) such that it was able to form a side-chain to main-chain hydrogen bond through its ε2 nitrogen atom and the oxygen atom of C246 in domain II(B). A hydrogen interaction from S429 and G245 in domain III(A) also strengthened this binding interface. Other interactions, such as the hydrophobic interactions made by P398 and W430 in domain III(A) and L244 in domain II(B), the hydrophilic interactions made by E481 in domain III(A) and S288 in domain II(B), and the side-chain to side-chain hydrogen bond between Q156 in domain I(B) and H490 in domain III(A), are also observed, but these interactions did not seem critical to the interface (Fig. S2C and Table 2).

### Functional characterization of ErbB2 ECD homodimer

**Figure 3:**
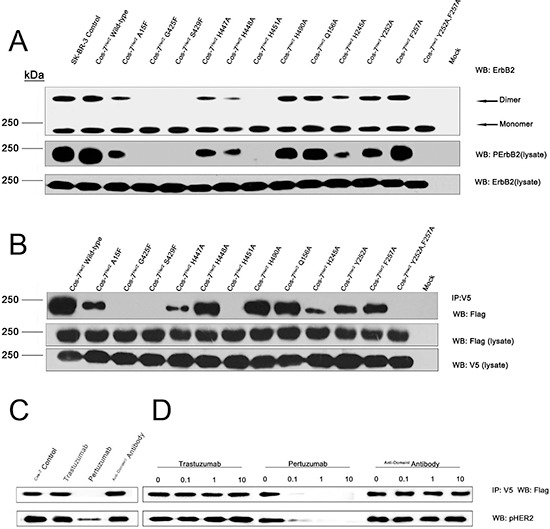
The impacts of key residues as well as the therapeutic antibodies on ErbB2 dimerization and phosphorylation **(A)** Crosslinking and immunoblotting assays for key residues of the homophilic ErbB2 interaction interface. **(B)** Co-immunoprecipitation assay for key residues of the homophilic ErbB2 interaction interface. **(C)** and **(D)** Pertuzumab, but not trastuzumab, sterically interferes with ErbB2 homodimerization in the co-immunoprecipitation assay.

To further investigate the important roles of the residues located at the dimer interface, we mutated these residues and verified their impacts on full-length ErbB2 dimerization and phosphorylation in COS-7 cells (Fig. 3A and 3B).

Among these interfacial residues, A15, G425, and S429 of ErbB2(A) hydrophobically interact with F257, T254, and L243 of ErbB2(B) and are positioned at the mouth of a C-shaped pocket. The replacement of these residues with phenylalanines is likely to narrow the C-shaped pocket and cause steric hindrance in the area. Consistent with this hypothesis, the G425F mutation caused the almost complete loss of dimerization and subsequent phosphorylation, and the A15F and S429F mutations also obviously impacted the formation of ErbB2 tyrosine-phosphorylated homodimers. H447 and H448 of ErbB2(A) interact with Y252 and F257 of ErbB2(B). The mutant H447A and double mutant Y252A/F257A significantly attenuated ErbB2 dimerization and phosphorylation. In contrast, the H448A, Y252A, and F257A single mutations exhibited only slight effects on ErbB2 dimerization and phosphorylation. H451 of the ErbB2(A) protomer makes a “face to face” parallel hydrophobic π bond and a hydrogen bond to the H245 and C246 of ErbB2(B) protomer. Substitution of H451 and H245 with alanines abolished the dimerization and autophosphorylation of ErbB2. Alanine mutants of residues Q156 and H490, which form an additional hydrogen bond in ErbB2 dimer, had no detectable impact on ErbB2 dimerization and phosphorylation, indicating their minor role in ErbB2 dimerization. From these results, G425, H447, the Y252/F257 pair, H451, and H245 were revealed to play the most essential roles in ErbB2 dimerization. Interestingly, Y252 and F257 in domain II(B) may facilitate heterodimerization with other ErbB molecules, perhaps even with ErbB monomers from different species, through hydrophobic interactions. In these heterodimers, Y252 and F257 of ErbB2 could interact with the corresponding residues in other monomers, such as ErbB1 residues Y246 and Y251 or dEGFR residues Y242 and Y247 [12, 18] (Fig. S3A, B and C).

**Figure 4:**
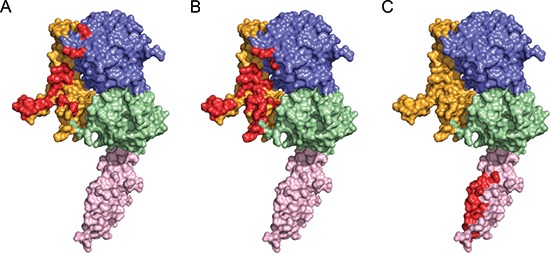
Comparison of the interface between ErbB2 homodimer and therapeutic antibodies ErbB2 from the complex structures is represented as a colored surface and the homodimieric interface on domain II **(A)** Pertuzumab binding interface **(B)** and Trastuzumab binding interface **(C)** highlighted in red. Residues 506–607(Domain IV) is modeling from PDB ID code 1N8Y.

Pertuzumab and trastuzumab are two clinically administered anti-ErbB2 antibodies with variable mechanisms of action. Pertuzumab binds at the dimerization arm [11], and trastuzumab's epitope is located in domain IV [6] (Fig. 4). Thus, we hypothesized that the binding of pertuzumab to ErbB2 would abolish the dimerization, but trastuzumab would not. Consistent with this hypothesis, the results of co-immunoprecipitation and the subsequent phospho-ErbB2 detection assay revealed that pertuzumab is able to prevent ErbB2 dimerization and phosphorylation, but trastuzumab cannot (Fig. 3C and 3D). These results support the critical role of domain II in ErbB2 homodimerization.

## DISCUSSION

It is widely accepted that the homo and/or heterodimerization of ErbBs [19, 20] switch on the phosphorylation of tyrosine residues in their ICDs, resulting in the activation of signaling associated with the SH2 (Src, homology region 2) and PTB (phosphotyrosine-binding) domains [21, 22]. These events lead to downstream signaling pathways, including the Ras (MAP) kinase, phospholipase Cγ (PLCγ), signal transducer and activation of transcription (STATs), and phosphatidylinositol-3 kinase (PI-3K) pathways [23, 24]. The ErbB1 ECD and its ligand, EGF, form a symmetric 2:2 complex [12], and a similar dimerization mode was observed for an independently determined structure of a 2:2 complex of ErbB1 and TGF-α [25]. In this receptor-mediated “back to back” model, two receptors bind to each other directly, and each of the two ligands binds to only one receptor. There is no contact between a given ligand and either the opposite receptor or the other ligand, and each of the receptors' two Cys-rich domains (domain II and IV) extend their dimerization arms to hold onto the body of the other.

The ligand-independent activation and self-association of ErbB2 occurs when it is overexpressed at the plasma membrane [26], and its ECD has been indicated to contribute to this dimerization process [14, 27]. Our structure revealed that the ErbB2 ECD plays an essential role in ErbB2 dimerization: two ErbB2 protomers directly bind to each other through an interaction between the dimerization arm and the C-shaped pocket. Although this “back to head” dimerization conformation can also be observation in the free status of ErbB2 [28] as well as the crystal of the ErbB2 in complexed with trastuzumab fab [6], which is consistent with our data that trastuzumab has minor effect on the homodimerization of ErbB2. However, the two papers which reported the structures did not discuss this binding interface, maybe for the reason that these interaction were binding with a symmetry molecule. The ErbB2 ECD homodimer structure is not symmetrical like that of the ErbB1 ECD-ligand(s) dimer complex. This difference is most evident at the dimer interface near the domain II amino terminus.

**Figure 5:**
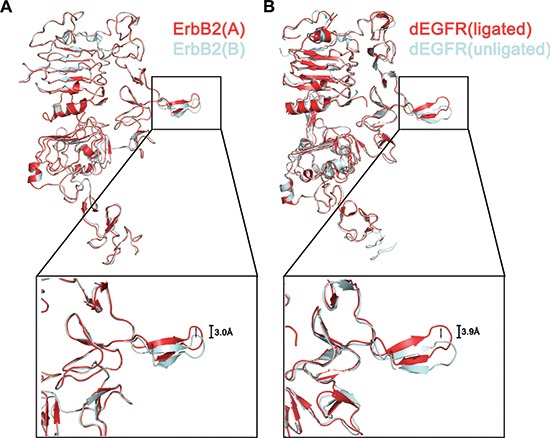
Conformational shift of dimerization arm in the ErbB2 ECD homodimer Superposition of an ErbB2 providing the dimerization arm (cyan) to the other ErbB2 (red) **(A)**. An overlay of one ligated molecule of dEGFR from a dimer (red) (PDB ID code 3ITF) on unligated dEGFR (cyan) (PDB code: 3I2T) **(B)**.

Another interesting observation is that domain II of the two protomers adopts a unique orientation caused by the homodimer interaction. Upon forming the homodimeric complex, domain II(B) “wedges” into the C-shaped pocket between domains I(A) and III(A), pushes them slightly apart, and then causes a substantial reorientation of domain II(A), which connects domain I(A) and III(A) (Fig. 5A). Analogous changes in domain conformation have been observed in the structure of the dEGFR dimer [29] (Fig. 5B). Domain II of an unligated (inactive) dEGFR is reoriented with a conformational shift of 3.9 Å when it binds to a ligand, leading to activation. We propose that ErbB2 may have a similar self-regulation mode by distorting domain II in only one of the two ErbB2 molecules in the dimer [the free protomer, ErbB2(A)] and does not allow formation of more extensive polymers during its function.

Previous study indicated that trastuzumab does not decrease ErbB2 homodimers despite its strong potency to drive ErbB2-overexpressing cells into quiescence, whereas pertuzumab is able to prevent ErbB2 homodimerization [30]. The structure of the ErbB2 homodimer provides an explanation for the inhibition of ErbB2-mediated signaling by pertuzumab. The pertuzumab binding epitope overlaps with the ErbB2 ECD homodimer interface (Fig. 4); thus, bound pertuzumab sterically prevents the dimerization arm from reaching the C-shaped pocket of the other ErbB2. In the ErbB2 ECD homodimer structure, the ErbB2 dimerization arm donates 20 residues to the dimeric interface, of which 17 residues (85%) are involved in the ErbB2-pertuzumab binding interface. In contrast, the anti-domain IV antibody, trastuzumab, seems to have little effect on ErbB2 homodimerization (Fig. 4). These studies provide new insights into the dimerization of ErbB2. These findings suggest a back to head dimerization architecture formed by the dimerization arm and a C-shaped pocket and reveal the key residues at the interface that are crucial for ErbB2 dimerization and phosphorylation. Our model not only provides a framework for understanding the molecular mechanism of EGFRs dimerization but also aids in the development of new therapeutic antibodies.

## MATERIALS AND METHODS

### Cell lines and antibodies

The human breast cancer cell lines SK-BR-3 and African green monkey SV40-transformed kidney fibroblast cell line (COS-7) were obtained from the American Type Culture Collection (ATCC, Manassas, VA). The cells were cultured in DMEM medium supplemented with 15% fetal calf serum in 5% CO2 at 37.8°C in a humidified incubator. Trastuzumab and Pertuzumab antibody was purchased from Roche Ltd.

### Construction, expression and purification

Residues 1–624 of the extracellular domain of ErbB2(ErbB2-ecd) was prepared as described previously [13], except that we used the pcDNA3.1(+) expressing vector (Invitrogen, Carlsbad, CA) and the FreeStyle 293 expression system (Invitrogen). The amino acid sequence of the antibody light chian V region is: QSALTQPASVSGSPGQSITISCTGTSSDVGGYNYVSW YQQHPGKAPKLMIYDVSKRPSGVSNRFSGSKSGNT ASLTISGLQAEDEADYYCSSYTSSSTLVFGGGTKLT VLG and amino acid sequence of the heavy chain V region is: EVQLVQSGAEVKKPGESLKISCKGSGYSFTSYWIGW VRQAPGQGLEWMGWISAYNGNTNYAQKLQGRVT MTTDTSTSTAYMELRSLRSDDTAVYYCAREGDGA FDYWGQGTLVTVSS. The recombinant antibodies were purified through protein A affinity chromatography from the serum-free culture supernate. The antibody concentrations were determined by absorbance at 280 nm, and the purity was confirmed through SDS–PAGE analysis and western blot.

The Fab fragment of the human anti ErbB2 domain I antibody for the crystallographic investigation was obtained through a papain digestion of an antibody. The digested protein sample was loaded onto a Protein A Sepharose 4 FF column (GE Healthcare). The Fab fragment eluted in the flow through was separated from the Fc fragment and further purified through ion-exchange chromatography using a Q-Sepharose FF column (GE Healthcare). The protein sample was concentrated to 20 mg/mL and then exchanged to a stock buffer containing 20 mM Tris–HCl (pH 8.0) and 300 mM NaCl. ErbB2-ecd was subsequently mixed with an excess of H218 Fab, and the complex was purified through gel-filtration chromatography (GE Healthcare). This complex was dialyzed against 10 mM Tris–HCl (pH 8.0) and 50 mM NaCl, and was concentrated to 25 mg/ml.

### Crystallization

Crystallization was performed at 291 K through the hanging-drop vapor-diffusion technique. The crystals were obtained by mixing 1 μl of the protein solution with an equal volume of a reservoir solution. The mixture drop was equilibrated against 500 μl of the reservoir solution. The crystals were obtained with a reservoir solution containing 0.1 M Ammonium acetate, 0.1 M BIS-TRIS pH 5.7, 13% w/v Polyethylene glycol 10,000, which reached the final dimensions of 100 × 100 × 100 μm^3^ with the best diffraction within two weeks. The crystals were then cryo-protected through soaking in a cryo-protectant comprising the reservoir solution and 10% glycol. The cryo-protected crystals were subsequently flash-cooled in liquid nitrogen, and then transferred into a dry nitrogen stream at 100 K for X-ray data collection.

### X-ray data collection, processing, and structure determination

The diffraction data for the ErbB2-Fab complex was collected at beamline BL17A (Photon Factory, Japan) with a resolution of 3.1 Å. The data were processed, integrated, and scaled using the HKL2000 package. The crystals belong to space group P1 with cell parameters *a* = 84.7 Å, *b* = 104.2 Å, *c* = 116.7 Å, *α* = 107°, *β* = 99°, *γ* = 111°. The statistics of all data collections and structure refinements are summarized in Table 1.

The ErbB2-Fab structure was solved through the molecular replacement method, which employs the crystal structures of ErbB2 and Pertuzumab Fab (PDB code: 1S78) as the initial searching model by using the program PHASER. The clear solutions in both the rotation and translation functions indicated the presence of two complex molecule, including two ErbB2 and two Fab molecules, in one asymmetric unit. This result is consistent with the Matthews coefficient and solvent content. The inconsistent residues were manually rebuilt in the program Coot under the guidance of the *Fo–Fc* and 2*Fo–Fc* electron density maps. The residues were refined in PHENIX, and the respective working R-factor and R-free decreased from 0.44 and 0.45 to 0.23 and 0.27, respectively, for all data from 50.0 Å to 3.1 Å. The refinement was monitored by calculating *R_free_* based on a subset containing 5% of the total reflections.

### ErbB2 mutagenesis and cos-7 transfections

ErbB2 Site-directed mutants (A15F, G425F, S429F, H447A, H448A, H451A, H490A, Q156A, H245A, Y252A, F257A or double mutants Y252A/F257A) were created through PCR. All wild-type and ErbB2 mutant constructs used in this study were epitope tagged at the C terminus with a FLAG sequence or a V5 sequence. All mutations were confirmed by automated DNA sequencing. COS-7 cells (1 × 10^6^ cells/10 cm dish) were transfected with 1 ug of ErbB2-wild-type-flag, ErbB2-mutant-flag DNA with or without ErbB2-wild-type-V5 DNA using Lipofectamine according to the manufacturer's recommendations (Invitrogen). We also use The human breast cancer cell lines SK-BR-3 cells as a positive control. Twenty-four hours post transfection, cells were serum starved overnight in 0.1% fetal bovine serum/DMEM at 37°C prior to treatment.

### Cross-linking and immunoprecipitation assays

Cells were cross-linked as described [14]. Generally, subconfluent cells were rinsed once with Hepes buffer (150 mM NaCl, 25 mM Hepes). BS3 (bis(sulfosuccinimidyl)suberate) (Pierce) was added to cells at a final concentration of 1 mM and incubated for 2 h at room temperature. After cross-linking the cells were rinsed with Tris saline and lysed in RPMI 1640 lysis buffer followed by immunoprecipitation with an HER2 antibody Ab8 (Neomarkers), as described [14, 15]. Immune complexes and whole cell lysates were subjected to SDS-PAGE and transferred onto nitrocellulose membrane with primary antibody for immunoblotting ErBB2(sc-7301; Santa Cruz Biotechnology, Santa Cruz, CA). The inhibition of homomeric association could not be attributed to the lack of receptor expression at the cell surface because indirect staining of transfected cells by whole cell lysates ErbB2 detection using western-blot showed that wild-type and mutant ErbB2 were expressed at comparable levels.

### Coimmunoprecipitation assays

Homodimer of ErbB2 was aslo evaluate in a coimmunoprecipitation assay, as describe [11, 16, 17]. Transfected cells were lysed in 1 ml RPMI lysis buffer (1% v/v DevelopTriton X-100, 1% w/v CHAPS, 10 mM HEPES (pH 7.2), in RPMI medium containing 0.2 mM PMSF, 10 ug/ml leupeptin, 10 U/ml aprotinin, and 1 mM Na_3_VO_4_). Homodimers were immunoprecipitated from 500 ul of lysate using mouse anti-V5 antibody(AbD Serotec) covalently coupled to agarose (Pierce ultralink) at 4°C for 2 h. Complexes were washed twice in lysis buffer and resuspended in SDS sample buffer and boiled. Samples were separated on a 4%–12% polyacrylamide gel (Novex) and electro-blotted onto nitrocellulose membranes. Blots were blocked in 10% BSA/TBST and probed with an anti-Flag antibody(F3165, Sigma) to detect ErbB2 followed by a peroxidase-conjugated anti-mouse secondary antibody (Amersham) To verify and normalize for expression of transfected constructs between experimental conditions, 50ug of cell lysate was checked by western blotting with anti-V5 antibody (AbD Serotec) or anti-Flag antibody(Sigma). Anti-mouse HRP-conjugated secondary antibody was used for visualization by enhanced chemiluminescence (ECL, Amersham Pharmacia Biotech).

### Phosphorylation assays

Ligand independent activation of ErbB2 or ErbB2 mutants was detected by a tyrosine phosphorylation assay using an anti phospho-ErbB2-Tyr1221/1222 antibody (2243; Cell Signaling)

## SUPPLEMENTARY FIGURES


