# Supplementary material for: Molecular architecture of the ErbB2 extracellular domain homodimer
Source: Oncotarget. 2015 Jan 30;6(3):1695–706. doi: 10.18632/oncotarget.2713 (PMC4359325; doi:10.18632/oncotarget.2713)
Supplement: Supplementary file 1 [file oncotarget-06-1695-s001.pdf]

SUPPLEMENTARY FIGURES

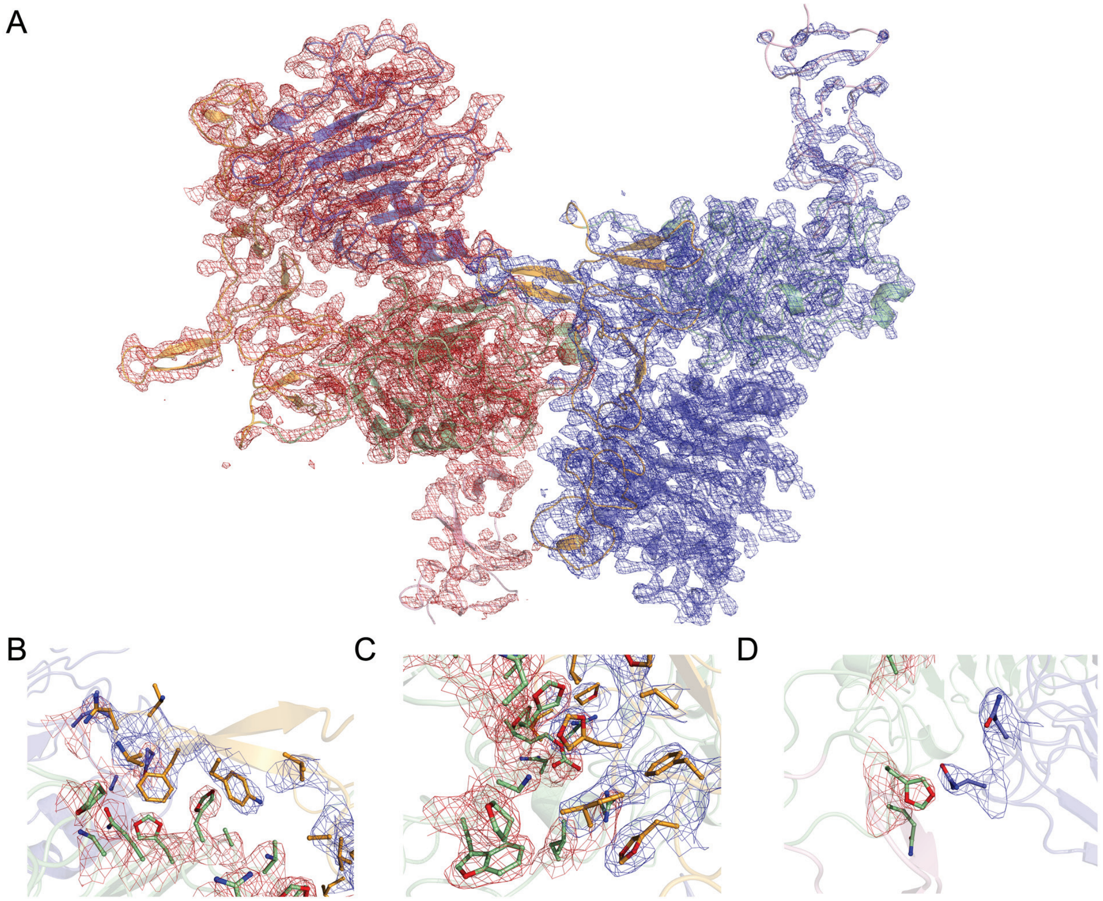

Supplementary Figure S1: Experimental density of the ErbB2 homodimer and the dimer interface at 3.1 Å resolution. Omit map of the ErbB2 homodimer **(A)** and interaction sites **(B–D)** is contoured at 2.0 σ. ErbB2 is shown as cartoon, while the side chains of interacting residues are represented as colored sticks.

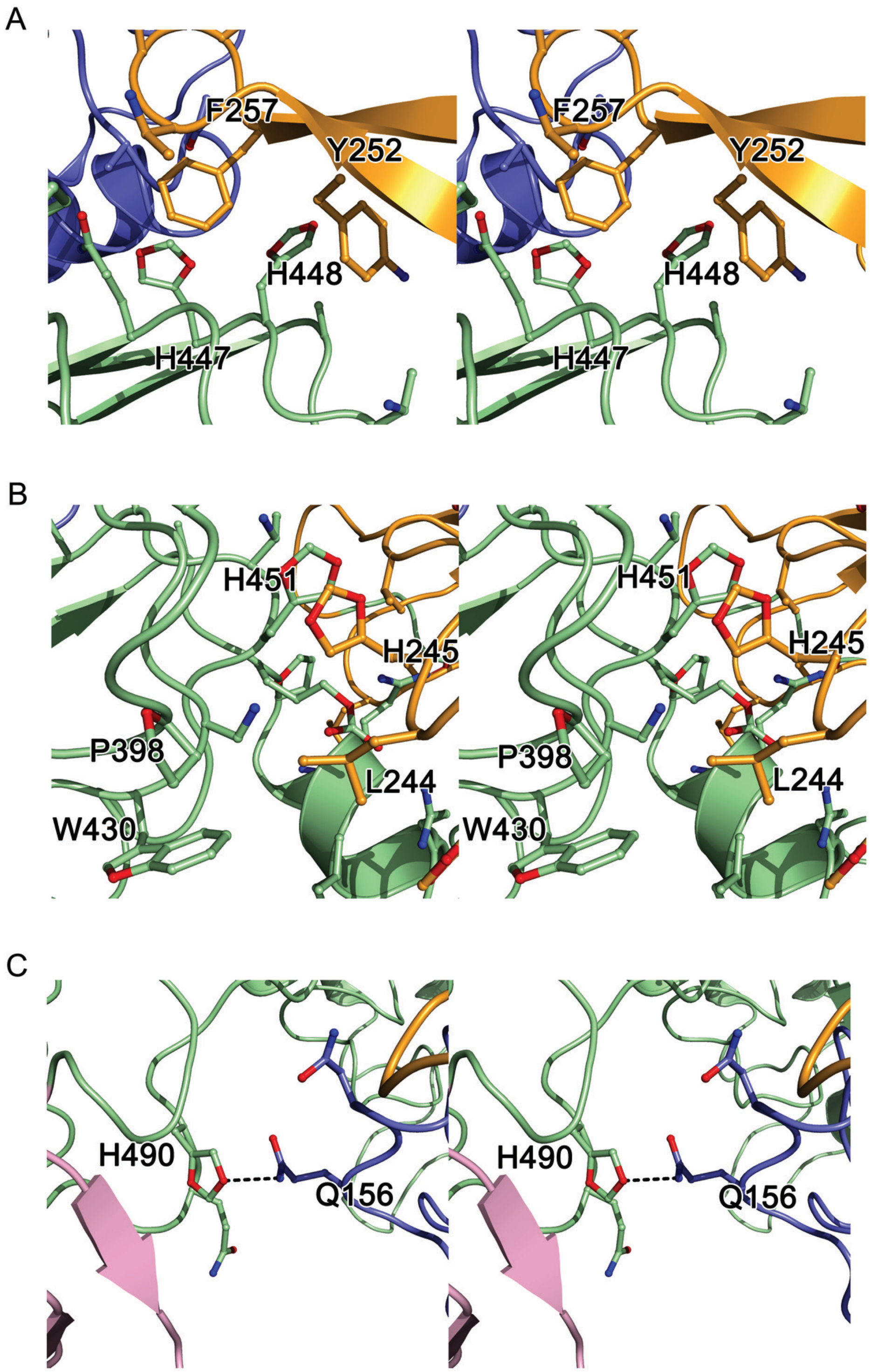

Supplementary Figure S2: Stereo view of Interactions between the two ErbB2s in the Dimer Interface. Only the side chains of interacting residues are shown. Dotted lines represent hydrogen bonds. **(A)** Interactions within the β-hairpin arm in Domain II. **(B)** Central role of H451 in Domain III. **(C)** Interaction within Domain III.

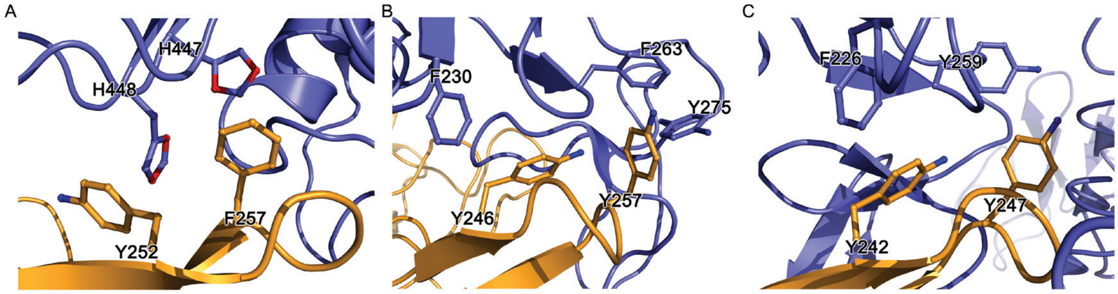

Supplementary Figure S3: Comparison of the hydrophobically interactions amony ErbBs. ErbB2 homodimer **(A)** ErbB1 homodimer **(B)** and dEGFR homodimer **(C)** are shown in figure and the dimerization arm is colored yellow.
